# At-home technique for debridement of nail plate thickening

**DOI:** 10.1016/j.jdcr.2024.07.002

**Published:** 2024-08-29

**Authors:** Eden Axler, Zachary Neubauer, Shari R. Lipner

**Affiliations:** Department of Dermatology, Weill Cornell Medicine, New York, New York

**Keywords:** debridement, hyperkeratosis, nail, nail thickening, onychodystrophy

## Introduction

Nail plate thickening is a common clinical finding that can be observed in many onychodystrophies, including retronychia, pincer nails, traumatic onychodystrophy, pachyonychia congenita, nail psoriasis, and onychomycosis. Patients often report difficulties with nail trimming, walking, and wearing shoes comfortably. They may experience pain and embarrassment associated with the appearance.^1^ Despite its prevalence, at-home treatment options for nail thickening are limited to softening agents, such as urea cream, or pumicing/filing. In office techniques, including avulsion, debridement, and camouflage can be painful, inconvenient, and/or costly.^2^ We describe a patient who used an at-home nail drill to treat his nail plate thickening.

## Case

A 62-year-old man, Fitzpatrick skin type II, presented to our specialty nail clinic with nail plate thickening involving all 10 toenails for the past 20 years. Physical examination was significant for nail plate thickening and xanthonychia of the toenails. Histopathology of the nail plate clipping was negative for hyphae, consistent with traumatic onychodystrophy, likely due to his frequent running. He had been prescribed a topical antifungal cream by a previous physician and reported using it for several months with no improvement in the nail thickening. Treatment with topical urea 40% cream for 2 months was ineffective in thinning his nails. Nail pumicing and filing were not performed. The patient complained of discomfort when wearing shoes, difficulty with nail trimming, and feeling ashamed about the appearance of his nails.

The patient began using an at-home nail drill twice monthly, which effectively thinned his nails to allow for efficient nail clipping, improvement in quality of life, and increased comfort while wearing shoes.

## Discussion

**Fig 1:**
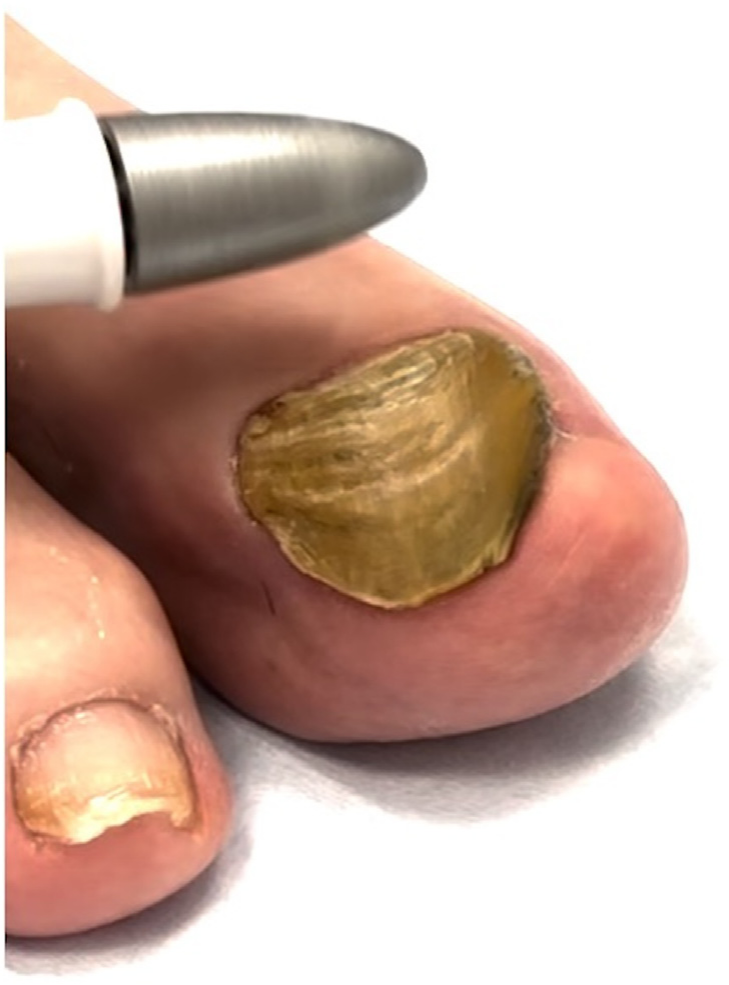
A 77-year-old man, Fitzpatrick skin type II, with retronychia. Bilateral great toenails with hyperkeratosis and xanthonychia before at-home nail drill use.

Nail plate thickening can be triggered by a variety of causes, ranging from trauma to structural abnormalities of the foot including hallux valgus, hammer and mallet toes, Morton toe, adductovarus fifth toe, hallux extensus, hallux limitus/rigidus, digiti flexi, foot-to-shoe incompatibility, hallux rigidus, halluxvalgus, and overlapping and underlapping toes (Fig 1).^1^^,^^3^ In a prospective study including 82 onychodystrophy cases, 92% of patients had a podiatric deformity and 48% had a history of nail trauma.^4^

Patients with traumatic onychodystrophy present with hyperkeratotic and discolored nails, typically accompanied by a reduction in nail plate translucency.^3^ Diagnosis requires exclusion of other conditions that can present similarly, including onychomycosis or nail psoriasis, and a nail plate clipping with histopathologic confirmation can be used to rule out these entities. When conducting a clinical evaluation, it is important to ask about any past nail trauma, types of footwear, exposure to occupational risks, or relevant medical conditions such as hemiplegia or neurologic issues. During the physical examination, it is important to inspect the foot for structural irregularities, including overlapping toes, Morton toe, and hallux varus, which could influence the development of nail plate thickening.

Treatment of traumatic onychodystrophy is challenging. Filing or pumicing may be attempted, but is often difficult to perform and ineffective for very thick nails. Topical urea 40% is the primary medical treatment for this condition, and the mechanism of action is to promote unfolding, solubilization, and dissociation of protein molecules in the nail plate, which facilitates the cleavage of disulfide linkages.^5^ Topical urea promotes nail plate softening and may also enhance transungual drug permeation.^6^ Urea 40% cream is applied directly to the nail plate nightly with occlusion, and may be used alone, or in conjunction with nail drilling twice monthly. In office nail plate debridement with drilling is not typically performed by dermatologists, although in podiatry, drilling is routinely used to manage thickened nails, onychomycosis, corns, and calluses, providing pain relief, improved treatment outcomes, and enhanced mobility. Surgical treatments for nail plate thickening are limited to case reports. Baran et al^7^ reported successfully surgically removing one-third of the distal nail matrix in a case report of a 47-year-old with Darier disease. Another case report describes application of phenol to the proximal nail matrix to permanently ablate the matrix.^8^ A prospective study demonstrating prosthetic nail resin application for 15 patients with retronychia reported improvement in nail appearance in 87% of patients.^2^

**Fig 2:**
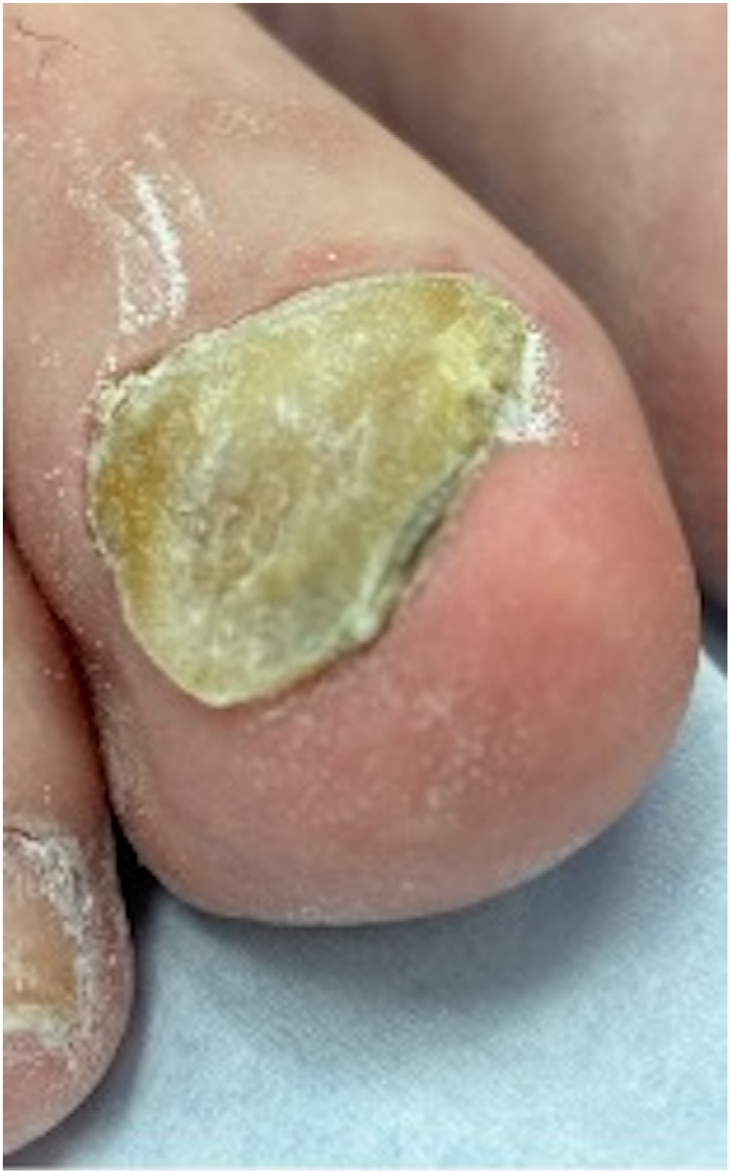
A 77-year-old man, Fitzpatrick skin type II, right great toenail with retronychia. Nail plate has been debrided with at-home nail drill and is significantly less hyperkeratotic.

At-home nail drills are a noninvasive, quick, painless treatment option for nail thickening. These drills are equipped with various sized drill bit attachments composed of sapphire and felt. The cylindrical drill bit removes unwanted layers of the nail, whereas fine drill bits are used for filing (Fig 2). The speed is adjustable, and some devices have a dust shield that collects scattered particles during the filing process (Supplementary Fig 1, available via Mendeley at https://data.mendeley.com/datasets/svzhm3w4f7/1). Using an at-home nail drill carries a low risk of infection for most individuals. Potential risks include accidental cuts or overthinning the nails if used too frequently (recommended use is once every 2 weeks). For patients who cannot reach their nails, have impaired sensation, or are at high risk of infection, we recommend seeing a podiatrist to avoid potential risks. The average cost of the top 50 devices on Amazon, (search term “at home nail drill,” accessed April 13, 2024) averaged $22.60 (range $8.99-$49.99, median $16.00). At-home nail drills provide a convenient, noninvasive solution for nail thickening, improving cosmesis and comfort affordably.

## Conclusion

Nail plate thickening presents a common clinical challenge across a broad range of onychodystrophies, impacting patient quality of life and causing discomfort. Current treatment options, including topical urea 40% cream and surgical interventions, often yield unsatisfactory outcomes, are invasive, or costly. An at-home nail drill offers a promising alternative as a noninvasive, cost-effective solution for nail thickening.

## Conflicts of interest

Dr Lipner has served as a consultant for Ortho-Dermatologics, Eli Lilly, Moberg Pharmaceuticals, and BelleTorus Corporation. Authors Axler and Neubauer have no conflicts of interest to declare.
